# Editorial: Cardiopulmonary support during catheter ablation of ventricular arrhythmias with hemodynamic instability

**DOI:** 10.3389/fcvm.2024.1450061

**Published:** 2024-07-09

**Authors:** Antonio Di Monaco, Gemma Pelargonio, Massimo Grimaldi

**Affiliations:** ^1^ Department of Cardiology, General Regional Hospital “F. Miulli”, Bari, Italy; ^2^ Department of Cardiovascular Sciences, Catholic University of the Sacred Heart, Rome, Italy; ^3^ Department of Cardiovascular Medicine, Fondazione Policlinico Universitario A. Gemelli IRCCS, Rome, Italy

**Keywords:** catheter ablation (CA), electrical storm, mechanical support devices, ventricular ablation, hemodynamic instability, ventricular arrhythmias

**Editorial on the Research Topic** Cardiopulmonary support during catheter ablation of ventricular arrhythmias with hemodynamic instability

Ventricular arrhythmias (VA) and electrical storm (ES) are often life-threatening determining haemodynamic impairment, cardiogenic shock and cardiac arrest. The mortality in these conditions rate up to 50% and, for this reason, restoration and maintenance of sinus rhythm is crucial (1).

Antiarrhythmic drugs are usually used as a first line therapy but they may be ineffective and may cause side effects (1). Catheter ablation (CA) is the treatment of choice for refractory VA and is useful to reduce ES recurrence (1).

Acute heart failure during CA in patients with complex substrates may occur in 11% of cases and is associated with an increased mortality (2). Mechanical circulatory support (MCS) may be beneficial in treating haemodynamically unstable VA, providing maintenance of organ perfusion and allowing for an accurate treatment of the arrhythmias (3–5).

Veno-arterial Extracorporeal Membrane Oxygenation is the preferred rescue MCS in patients with ES undergoing CA. However, a high mortality rate in the short-term is reported (range from 38 to 76%) despite a successful CA (3). On the other hand, preemptive MCS in selected high-risk patients might reduce the risk of acute heart failure and death during follow-up. The identification of patients at high risk of periprocedural acute heart failure is a matter of priority, since preemptive MCS can improve CA safety (3).

Large randomized clinical trials comparing different devices and different ablation strategies in the setting of ES are not available; prospective multicentre data on preemptive MCS before CA are missing too.

The Research Topic “*Cardiopulmonary Support During Catheter Ablation of Ventricular Arrhythmias with Hemodynamic Instability*”, with four reviews, reports important aspects of using the MCS during CA in patient with unstable VA.

The study of Loardi et al. summarizes the features, implantation techniques, and results of current devices used for adult MCS. In particular, the study analyzes in detail all the devices available for MCS both for short-term support (devices used to support ablation procedures) but also devices for long-term support in patients with advanced heart disease as a bridge to cardiac transplantation. This review allows the reader to know in detail all the devices for MCS and their usefulness in the clinical practice.

The systematic review and meta-analysis of Shalganov et al. provides the reader with useful aspects to understand the importance of the ablation procedure for VA in terms of efficacy and safety and, above all, provides an analysis of all the studies that have evaluated the early ablation of VA. In particular, the findings of this study underline the use of early ablation in patients with structural heart disease and recurrent VA with the purpose of reducing ICD shocks, arrhythmic storm and hospitalizations for cardiovascular reasons, without increasing the complications related to the treatment.

Spartalis et al. reported all the studies regarding the use of MCS during CA of VA in patients with cardiogenic shock. The authors stressed the importance to perform an accurate pre-procedural risk-stratification of patients before CA of VA. Some patients performing CA in a stable hemodynamic status may encounter periprocedural acute heart failure which has a significant influence on subjects' mortality. All the individuals at high risk for ablation should be safeguarded with preemptive MCS regardless of the pathophysiology of the cardiovascular disorder, the CA technique and their hemodynamic condition at the beginning of the procedure. The goals of MCS must be to restore an acceptable circulatory state, facilitate CA and bridge individuals to target therapy for cardiac failure.

Finally, De Potter et al. reported a mini-review focused on the non-invasive neurological monitoring to enhance MCS-assisted VA ablation. By enhancing cardiac output, MCS may facilitate conditions for ventricular mapping during the arrhythmia that would otherwise be incompatible with survival. However, it is currently unknown the optimal mean arterial pressure to maintain end-organ perfusion in presence of nonpulsatile flow. Near-infrared oxygenation monitoring during MCS provides assessment of critical end-organ perfusion during VA, ensuring a continual assurance of adequate brain oxygenation during CA procedure. A careful monitoring through an electroencephalogram also provides additional information improving the assessment of complete brain recovery after VA termination. This focused review introduces practical case scenarios for such an approach showing how to reduce the risk of ischemic brain injury during these ablation procedures.

To summarize, the current Research Topic for Frontiers in Cardiovascular Medicine provides the reader with a comprehensive overview of the importance of MCS during CA of unstable VA.

Mechanical circulatory support is an effective treatment of cardiogenic shock due to VA. It is useful to preemptively support high-risk patients who require a CA for hemodynamically unstable VA and to ensure adequate cerebral perfusion during the procedure. An adequate brain oxygenation is crucial during these high-risk procedures to avoid neurological damages and its monitoring should be part of the routine setting during these complex ablations. MCS might be continued in the post procedure phase to wean the patient slowly allowing the heart to recover from stunning related to hemodynamic instability and to the energy delivered with ablation and DC shocks

Large randomized clinical trials comparing different devices and different ablation strategies for unstable VAs are needed to improve safety and efficacy in such setting.
